# Green Synthesis of Silver Nanoparticles from *Diospyros villosa* Extracts and Evaluation of Antioxidant, Antimicrobial and Anti-Quorum Sensing Potential

**DOI:** 10.3390/plants11192514

**Published:** 2022-09-26

**Authors:** Oluwatosin Temilade Adu, Farzana Mohamed, Yougasphree Naidoo, Temitope Samson Adu, Hafizah Chenia, Yaser Hassan Dewir, Hail Rihan

**Affiliations:** 1 Department of Biological Sciences, School of Life Sciences, College of Agriculture, Engineering and Science, Westville Campus, University of KwaZulu-Natal, Private Bag X54001, Durban 4041, South Africa; 2 Microbiology, School of Life Sciences, College of Agriculture, Engineering and Science, University of KwaZulu-Natal, Private Bag X54001, Durban 4041, South Africa; 3 Department of Physiological Sciences, Obafemi Awolowo University, Ile Ife 220005, Nigeria; 4 Plant Production Department, College of Food & Agriculture Sciences, King Saud University, Riyadh 11451, Saudi Arabia; 5 School of Biological Sciences, Faculty of Science and Environment, University of Plymouth, Drake Circus, Plymouth PL4 8AA, UK; 6 Phytome Life Sciences, Launceston PL15 7AB, UK

**Keywords:** DPPH, electron microscopy, nanoparticles, quorum sensing inhibition

## Abstract

The biosynthesis of silver nanoparticles (AgNPs) from *Diospyros villosa* leaves and stem bark extracts is described. The stem bark AgNPs of *D. villosa* synthesized at 80 °C (S80) showed good scavenging activity with a lower IC_50_ value of 8.75 µg·mL^−1^ compared to ascorbic acid (9.58 µg·mL^−1^). The total phenol content of the S80 AgNPs was measured and found to be 10.22 ± 0.14 mg.g^−1^ gallic acid equivalence (GAE). Bacterial growth inhibition (% GI) and violacein inhibition (% VI) of 10.08% and 58.83%, respectively, was observed against *C.* *subtsugae* CV017 with leaf AgNPs synthesized at 80 °C (L80) at 80 μg·mL^−1^. Stem bark AgNPs synthesized at room temperature (SRT) also indicated % GI of 13.83% and % VI of 65.97% against *C. subtsugae* CV017 at 160 μg·mL^−1^. Leaf AgNPs of *D. villosa* synthesized at room temperature (LRT), showed % GI of 29.07% and % VI of 56.53%, respectively, against *C. violaceum* ATCC 12472 at 320 μg·mL^−1^. The L80 and SRT at 160 μg·mL^−1^ and LRT at 320 μg·mL^−1^ may be considered as potential QS inhibitors following their activity against *C. subtsugae* CV017 and *C. violaceum* ATCC 12472, respectively. Therefore, *D. villosa* represents a potential source of antioxidants as well as an anti-quorum sensing therapeutic candidate for the control of Gram-negative bacterial infections.

## 1. Introduction

Plant biomolecules are well reported for their limiting effects on infectious disease [1,2,3] and their increasing antimicrobial activities have resulted in a continuous search for new plant-based compounds with diverse modes of activity for therapeutic purposes. The commonly used antibiotics exhibit bactericidal effects either by blocking bacterial enzyme activities, thereby rendering the bacteria susceptible to the antibiotic effect, or by attacking the bacteria directly [4]. The reduced potency of some antibiotics is becoming more obvious with recurrent ailments and disease. The pathogens have been shown to exhibit antibiotic resistance. Quorum sensing (QS) is an intercellular communication pathway in bacteria which involves synchronized genetic expression in response to cellular concentration [5]. It has been identified as a promising target for the detection of anti-virulence compounds against bacteria [6,7].

Green synthesis of nanoparticles (NPs) has been extensively utilized in recent years in the field of nanotechnology [8,9,10]. Among other materials, the synthesis of silver nanoparticles (AgNPs) has gained popularity due to its potent antimicrobial activity against microbes [11,12]. The biosynthesized AgNPs have greater antibacterial advantages compared with their antibiotic counterpart [13,14]. The variety of AgNP mechanisms of action include halting the replicative functions of bacterial cells by interrupting the membrane permeability, resulting into cell apoptosis by allowing the weakening of cell development [15]; attachment with protein functional groups leading to protein denaturation and apoptosis; inhibition of DNA duplication; cell membrane disruption; as well as leakage of bacterial cellular content with respect to protein denaturation and cell death [16]. Although there are some reports on QS and biofilm inhibition by NPs synthesized from plants [17,18,19], there is a need for further investigation of traditional plants being utilized for medicinal purposes but with no scientific documentation.

The synthesis of nanoparticles with the help of organic products have gained scientific attention due to its safety for living organisms and eco-friendliness [20]. Plants are suitable for the synthesis of AgNPs due to their appreciable amounts of phytochemicals with stronger antioxidant properties than reactive metallic ions [21,22]. Herbal plants are used to treat various ailments, including diseases caused by pathogens [23,24,25]. *Diospyros villosa* (*D. villosa*) is a plant which naturally occurs in South Africa. It is a perennial, bushy evergreen plant with a height range 1–4 m. The roots of the plant are used as a purgative remedy for gastrointestinal complications [26], as a toothbrush and to treat oral infection [27]. The medicinal efficacy of various plants found in the genus *Diospyros* for the treatment of illnesses like fever, whooping cough and diarrhea [28]. To the best of our knowledge, a single and not comprehensive report implicated naphthoquinones and triterpenes in the root extracts of *D. villosa* [27]. In addition, there is no scientific information on the use of the leaves and stem bark extract of *D. villosa*.

This research study was geared towards assessing the potential use of *D. villosa* leaves and stem bark extracts as bio-reduction agents for AgNP formation. The objectives were to characterize AgNPs synthesized from *D. villosa* leaves and stem bark extract, to determine their antioxidant potential and explore their quorum sensing inhibitory activity using *Chromobacterium* spp. biosensor systems.

## 2. Materials and Methods

### 2.1. Chemicals and Reagents

For the experiment, 2,2-Diphenyl-1-picrylhydrazyl (DPPH), gallic acid, acetic acid, Gentamicin and Ciprofloxacin were purchased from Sigma-Aldrich (St. Louis, MI, USA). Mueller–Hinton agar, hexane, methanol and chloroform were obtained from Merck Chemical Co. (Durban, South Africa).

### 2.2. Plant Collection

Fresh samples of mature leaves and stem bark of *D. villosa* were collected from KwaZulu-Natal, Durban, South Africa (29°84′33.6″ S, 31°4′12″ E). The plant was identified and a voucher specimen was deposited in the Ward Herbarium (01/18257) of the School of Life Sciences, University of KwaZulu-Natal. The collected plant parts (leaves and stem bark) were air-dried and pulverized into fine powder. The powdered samples were kept in a cool dry place for extraction purposes.

### 2.3. Plant Extraction

Powdered samples (8 g) of each plant part were heated to a temperature of 40 °C with 100 mL of Millipore ^TM^ water for 15 min. The solution was then filtered through Whatman No. 1 paper. The filtrate was further evaporated to dryness under reduced pressure at 40 °C in a rotary evaporator. The crude aqueous extracts were stored at 4 °C and used within 48 h.

### 2.4. Synthesis of Silver Nanoparticles (AgNPs)

The method described by Bodede et al. [29] was used in the synthesis of AgNPs. A prepared 5 mL aliquot of the plant extracts was thoroughly mixed with 50 mL of 1 mM silver nitrate (AgNO_3_). The resulting solutions were vigorously shaken together for 10 min to allow formation of the nanoparticles. The solutions were differently incubated on the benchtop at room temperature (25 °C) and at 80 °C using a water bath for 1 h (Table 1). The formation of NPs was indicated by dark brownish coloration of the solution. The color intensity of the solution was closely monitored and the container bearing the solution was removed once the color was observed to be completely bright. This was done to prevent the formation of NP aggregates which could occur almost as soon as the deepest brownish color was observed [30]. The solution was later stored in a dark environment to prevent the reduction of silver ions (Ag^+^) to Ag^0^ in the presence of sunlight [31,32]. Similarly, 5 mL of distilled water was added to a 50 mL aliquot of aqueous 1 mM AgNO_3_ as a control and the same procedure as described above was carried out. All analyses were carried out in triplicate. The obtained synthesized nanoparticles are as represented in the Table 1:

### 2.5. Quantification of AgNPs

The AgNP solutions obtained from the *D. villosa* leaves and stem bark at different temperatures (room temperature and at 80 °C) were centrifuged for 2 h at 1650× *g* at 4 °C using pre-weighed microcentrifuge tubes in an Eppendorf microcentrifuge (5804/5804 R, USA). The resulting supernatants from the AgNP solutions were decanted and the insoluble residues were resuspended in 20 mL of distilled water. This suspension was centrifuged three more times to remove inert substances. Samples were subsequently oven-dried for a period of 24 h at 40 °C. The microcentrifuge tubes were weighed again to calculate the yield of the synthesized AgNPs.

### 2.6. UV-Vis Spectra Analysis

A fractional portion of the AgNP yield was intermittently harvested to ascertain that the bio-reduction of Ag^+^ in the solution was fully completed. The aliquoted samples were diluted with 2 mL of distilled water. UV-2600 visible (Vis) spectra analysis was conducted using a spectrophotometer (Shimadzu, Tokyo, Japan) and assessing a range of wavelengths between 200 and 800 nm, with a resolution of 1 nm. The corresponding peaks were observed and recorded.

### 2.7. Fourier Transform Infrared (FT-IR) Analysis

FT-IR analysis was utilized for the detection of embedded biomolecules which are potentially accountable for the reduction and stability of AgNPs, as well as the surrounding state of the ligands used as a capping agent on top of the NPs [33]. FT-IR was carried out after bio-reduction by scraping off the residue that was attached to the capping ligand. The dehydrated nanoparticle was further analyzed to identify the functional groups of the biosynthesized AgNPs. The infrared spectra for the samples were achieved using spectrophotometer (Perkin-Elmer 100 FT-IR, Wellesley, MA, USA) which is further equipped with ATR testing accessory.

### 2.8. Scanning Electron Microscopy (SEM) and Energy Dispersive X-ray (EDX) Analysis

Approximately 40 μL of the AgNP samples were placed on the glass connected to a brass stub having carbon tape with adhesive strip on both sides. The affixed sample was allowed to dry with the aid of a mercury lamp for 60 min. Quorum (Q15OR ES) module sputter coater (vacuum of 0.1 Torr for 2.5 min) was further placed twice over the sample for a 10 min period. The morphological examination of the sample was made possible with the aid of a Zeiss Ultra-Plus field emission gun scanning electron microscope (FEG-SEM, Carl Zeiss, Munich, Germany) with acceleration voltage value of 5 kV [34]. The identified features were digitally captured by the NIS-D image software (AzTec analysis software, V. 1.2, Oxford Instruments, High Wycombe, UK). The Zeiss Ultra-Plus with an energy dispersive X-ray (EDX) spectrometer and attached to Astronomical Thermal Emission camera was also used to determine the elemental composition of the sample at an acceleration voltage of 20 kV.

### 2.9. Transmission Electron Microscopy (TEM)

The particulate nature (size and shape) of the biosynthesized AgNPs was determined using the transmission electron microscopy (TEM). A single drop of the AgNPs was deposited on a formvar-coated copper grid and allowed to dry for 10 min. The captured images were viewed using the Joel TEM 1010 (Joel, Tokyo, Japan) at 200 kV [35].

### 2.10. DPPH Scavenging Activity

The free radical scavenging ability of the biosynthesized AgNPs was tested and measured against DPPH (2,2-diphenyl-1-picrylhydrazyl) [36]. A 3 mL mixture of 0.004% DPPH solution in 95% ethanol, 0.1 mL of AgNPs and different concentrations of vitamin C was prepared. The prepared mixture was sonicated thoroughly and eventually made static for a period of 30 min. The strength of DPPH decolorization was confirmed at an absorbance of 517 nm. The control was also prepared using 0.1 mL of each constituent with distilled water utilized as a replacement of AgNPs or ascorbic acid. The percent inhibition of DPPH by the samples was determined by comparing both the absorbance values of the control and that of experimental samples. The higher the absorbance, the lower the scavenging ability [16]. The percentage DPPH radical scavenging activity was determined as thus:

$$\frac{Absorbance\;of\;Control-Absorbance\;of\;test\;samples}{Absorbance\;of\;control}\;\times \;100$$

### 2.11. Ferric Reducing Antioxidant Potential (FRAP) Assay

The Ferric Reducing Potential assay was carried out according to the procedure described by Juntachote and Berghofer [37]. One milliliter of multiple concentrations (15, 30, 60, 120 and 240 μg·mL^−1^) of AgNPs was prepared in triplicate. Each extract was also mixed with 25 mL of 0.2 M phosphate buffer (pH = 6) and 2.5 mL of 1% *w/v* potassium ferricyanide. The prepared mixture was incubated at 50 °C for 20 min and further mixed with 2.5 mL of 10% trichloroacetic acid. A 2.5 mL volume was further diluted twice with distilled water. Then, 0.5 mL of 0.1% ferric trichloride was eventually introduced into each AgNP mixture and incubated for 30 min. The absorbance of each prepared solution was the measured at 700 nm. The positive control used in this experiment was gallic acid. The concentration of AgNP producing 50% absorbance (IC_50_) was extrapolated from the graph where absorbance was plotted against the concentrations of the extract. Results were generated as follows:

$$\mathrm{Scavenging}\;\mathrm{effect}\;(\%)=[\frac{Absorbance\;of\;Control\;at\;700\;nm-Absorbance\;of\;sample\;at\;700\;nm\;}{Absorbance\;of\;control\;at\;700\;nm}]\;\text{×}\;100$$

### 2.12. Total Phenolic Content (TPC)

Total phenol content was determined using the colorimetric Folin–Ciocalteu assay with minor modifications [38,39]. Each AgNP (0.1 mL) at multiple concentrations (15, 30, 60, 120 and 240 μg·mL^−1^) was mixed with 3 mL of distilled water. A freshly prepared Folin–Ciocalteu reagent (0.5 mL) was added to each AgNP sample. The mixture was incubated for 3 min at room temperature. This was followed by the addition of 2 mL of 20% sodium carbonate and incubated further at room temperature for 30 min. The total phenolic content was measured at 725 nm using a spectrophotometer. Gallic acid was used as the positive control. The total phenol concentration was expressed as mg of gallic acid equivalents (GAE)/g of sample AgNPs.

### 2.13. Antimicrobial Susceptibility Test

The Kirby–Bauer disc diffusion assay was used to carry out the antimicrobial susceptibility testing. Four Gram-negative microorganisms, viz., *Escherichia coli* ATCC 25922, *Escherichia coli* ATCC 35218, *Pseudomonas aeruginosa* ATCC 27853 and *Klebsiella pneumoniae* ATCC 700603, as well as seven Gram-positive microorganisms: *Enterococcus faecalis* ATCC 29212, *Enterococcus faecalis* ATCC 51299, *Staphylococcus aureus* ATCC 29213, *Staphylococcus aureus* ATCC 33591, *Staphylococcus aureus* ATCC 43300, *Staphylococcus aureus* ATCC 700698, and *Staphylococcus epidermidis* ATCC 12228, were grown at 37 °C overnight on Mueller–Hinton (MH) agar plates. Inocula equivalent to a 0.5 McFarland were used to swab the surface of the MH plates [40]. Thereafter, blank discs were impregnated with 100 μg and 200 μg of the respective AgNPs and placed on the swabbed MH agar plates. Agar plates were then incubated for 24 h at 37 °C. Ciprofloxacin (CIP5) and Gentamicin (GN10) were used as the antibiotic controls. Following incubation, samples exhibiting zone diameters ≥ 16 mm were regarded as being strong antimicrobials, zone diameters between 11 and 15 mm were regarded as possessing intermediate activity and zone diameters ≤ 10 mm were considered weak antimicrobial agents.

### 2.14. Qualitative Quorum Sensing Inhibition

Qualitative evaluation of QS inhibition by the AgNPs was carried out based on their capacity to prevent the formation of violacein (purple in color) by *C. subtsugae* CV017 and *C. violaceum* ATCC 12472 [40]. Lack of purple pigment formation was an indication of violacein inhibition. About five milliliters of semi-solid Luria–Bertani (LB) agar was inoculated with 150 μL of the respective *Chromobacterium* biosensor strains that had been grown overnight at 30 °C in LB broth. The agar–culture mixture was then poured on pre-warmed LB agar plates and allowed to solidify. Thereafter, blank discs impregnated with 100 μg and 200 μg of the respective AgNPs were positioned on the agar plates and further incubated overnight at 30 °C. Discs impregnated with 100 μg and 200 μg of vanillin were used as the positive QS inhibition control. Following incubation, opaque zones indicative of QS inhibition and clear zones indicative of bactericidal activity were recorded.

### 2.15. Quantitative Quorum Sensing Inhibition

The QS inhibition of the AgNPs was quantified using *C. violaceum* ATCC 12472 and *C. subtsugae* CV017 as the indicator microorganisms [40]. One hundred microliters of an overnight culture of each biosensor were inoculated into 3 mL of LB broth and incubated at 30 °C with increasing concentrations of each AgNP sample, i.e., 0; 20; 40; 80; 160 and 320 μg·mL^−1^. For this assay, growth (OD_600_ nm) and violacein production (OD_560_ nm) was determined following overnight incubation at 30 °C. One milliliter of the overnight culture was then centrifuged at 13,000× *g* for 10 min to precipitate insoluble violacein. The obtained supernatant was decanted and pellets were resuspended in 1 mL dimethyl sulfoxide (DMSO) [30,41]. The resuspended solution was centrifuged again at 13,000× *g* for 10 min. Violacein was measured at OD_560_ nm using the Glomax Multi+ Detection System (microtiter plate reader) (Promega) [40].

The percentage violacein inhibition = $\frac{Control\;OD_{560}\;nm-Test\;OD_{560}\;nm}{Control\;OD_{560\;}\;nm}$ × 100 [40,42].

If the % growth inhibition was ≥40% of the positive, untreated control, the extract was considered to have growth inhibitory activity and not considered to be a good quorum sensing inhibition (QSI) indicator, which was indicated by % violacein inhibition ≥ 50%.

### 2.16. Statistical Analysis

Data are presented as mean ± SEM. Statistical analysis was performed using GraphPad Prism version 5 (Graph Pad Software Inc., San Diego, CA, USA). All outcomes were compared with control using analysis of variance (ANOVA) followed by Bonferroni post hoc analysis. Effects were considered statistically significant at *p* ≤ 0.05.

## 3. Results

### 3.1. Synthesis and quantification of AgNPs

**Figure 1:**
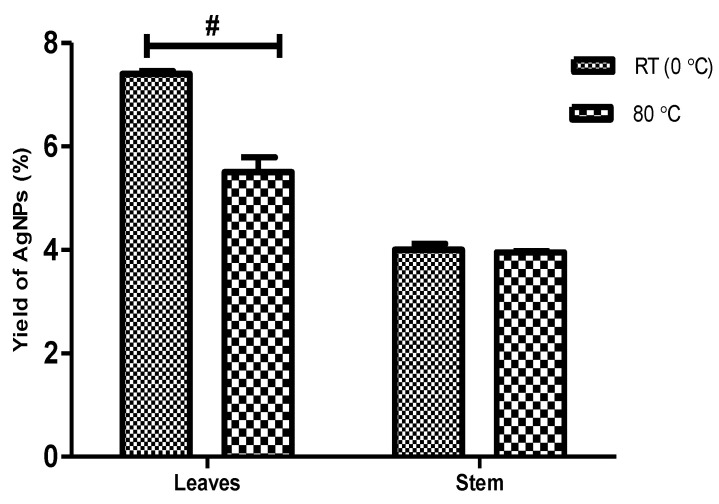
Percentage yield of AgNPs obtained from leaves and stem bark of *D. villosa* at room temperature (RT) and at 80 °C. F_(1,8)_ = 33.94. ^#^ (Leaves RT vs. Leaves 80 °C), *p* < 0.001.

The percentage yield of biosynthesized AgNPs from *D. villosa* leaves and stem bark are presented in Figure 1. The two-way ANOVA showed that there was a significant interaction between the temperature and variation in the parts of the plant (F_(1,8)_ = 33.94, *p* < 0.001). The percentage yield of AgNPs using the leaves extract at RT (LRT) was found to be significantly higher compared to that of synthesis at 80 °C (L80) (*p* < 0.001). The percentage yield of AgNPs using stem bark at RT (SRT) was not to be statistically different compared to that of 80 °C (S80).

### 3.2. UV-Visible Spectra Analysis

**Figure 2:**
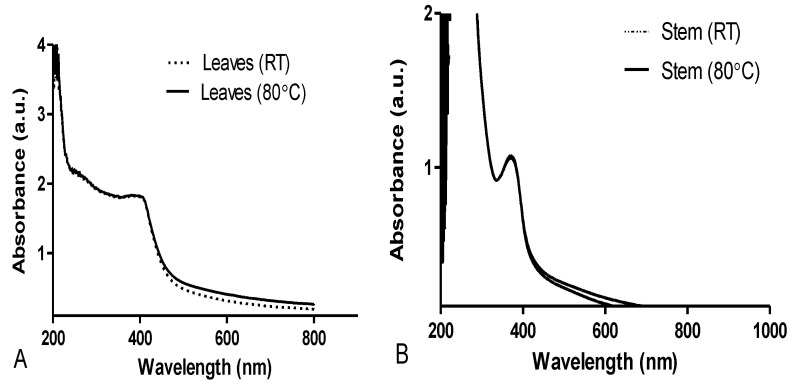
UV-visible spectra of *D. villosa* leaves (**A**) and stem (**B**) AgNPs at room temperature (RT) and at 80 °C.

For the biosynthesized AgNPs using leaves extract at RT and at 80 °C, the absorption peaks were found at 424 nm and 417 nm, respectively (Figure 2A). Absorption peaks were, however, observed at 367 and 369 nm for the biosynthesized AgNPs using stem bark extracts at RT and at 80 °C, respectively (Figure 2B). It was also observed that AgNPs biosynthesized from the leaves extract produced higher absorption peaks at RT compared to 80 °C. Similarly, the AgNPs synthesized from the leaves had higher intensities compared to AgNPs from the stem bark extract.

### 3.3. Fourier Transform Infrared (FT-IR) Analysis

**Figure 3:**
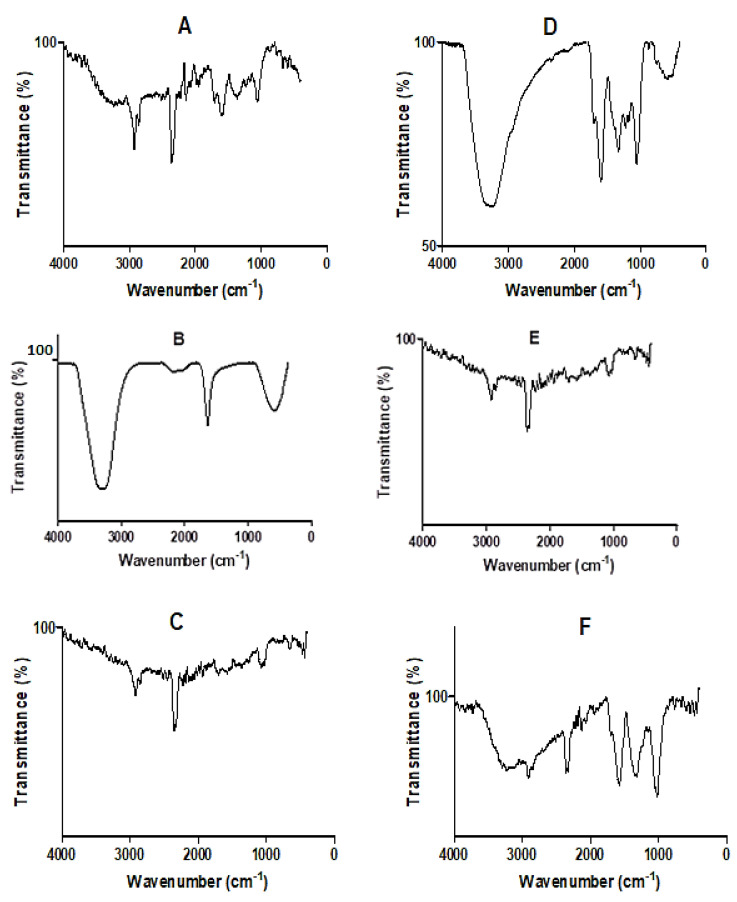
FT-IR spectra of crude extracts and the biosynthesized AgNPs of *D. villosa* plant. (**A**) Crude aqueous leaf extract; (**B**) leaves AgNPs synthesized at room temperature (LRT); (**C**) leaves AgNPs synthesized at 80 °C (L80); (**D**) crude aqueous stem bark extract; (**E**) stem bark AgNPs synthesized at room temperature (SRT); and (**F**) stem bark AgNPs synthesized at 80 °C (S80).

The FT-IR spectra of *D. villosa* leaves and stem bark and their corresponding nanoparticles are presented in Figure 3A–F. The spectra showed the vibrational frequencies of different functional groups found in both the respective crude extracts and in the AgNPs. The spectrum of the aqueous extract of the leaves and leaves AgNPs synthesized at 80 °C, as well as stem bark AgNPs synthesized at both room temperature and 80 °C, showed characteristic absorption bands for C-H stretching at 2929.37, 2937.87, 2937.87 and 2926.54 cm^−1^ (Figure 3A,C,E,F). Similarly, a strong O-H stretching band at 3369.91 and 3287.75 cm^−1^ was observed in the crude stem extracts and stem bark AgNPs at 80 °C (Figure 3D and 3F). There was also a strong absorbance band for carbonyl (C=O) stretching band at 1606, 1609.17 and 1602.09 cm^−1^ in both the crude leaves and stem bark extracts, as well as stem bark AgNPs synthesized at 80 °C (Figure 3A,D,F). It was also observed that a weak absorbance band for alkynes appeared within the range of 2365 and 2371.26 cm^−1^ in the AgNPs synthesized at RT, crude leaf extract and stem bark AgNPs at 80 °C.

### 3.4. Scanning Electron Microscopy (SEM) Analysis

**Figure 4:**
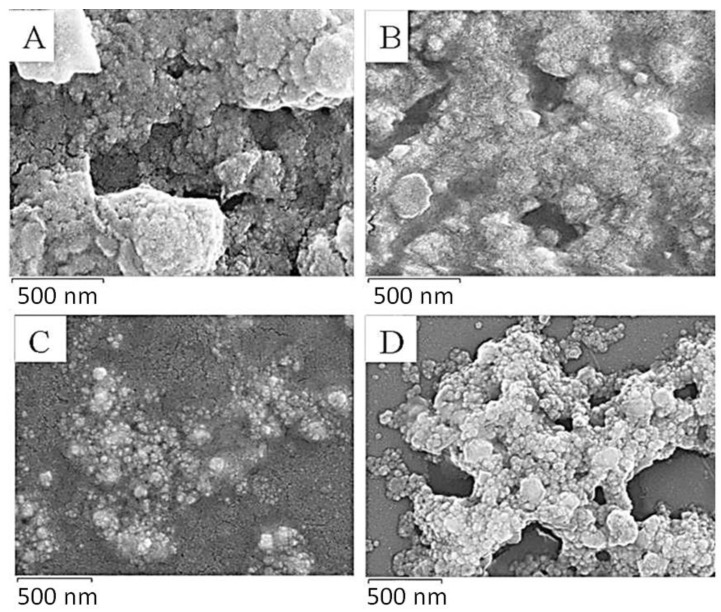
SEM images of *D. villosa* leaves (**A**,**B**) and stem bark (**C**,**D**) AgNPs synthesized at room temperature and at 80 °C, respectively.

The SEM images of the *D. villosa* leaves and stem bark AgNPs are presented in Figure 4. The images showed that the NPs of the *D. villosa* leaves synthesized at room temperature and at 80 °C were agglomerated and widely distributed. Meanwhile, the NPs of the stem bark extract (at 80 °C) were of similar sizes, spherical and well distributed (Figure 4D). The spherical morphology of the nanoparticles synthesized from the stem bark extract were more distinct compared to those synthesized from the leaves extract.

### 3.5. Energy Dispersive X-ray (EDX) Analysis

**Figure 5:**
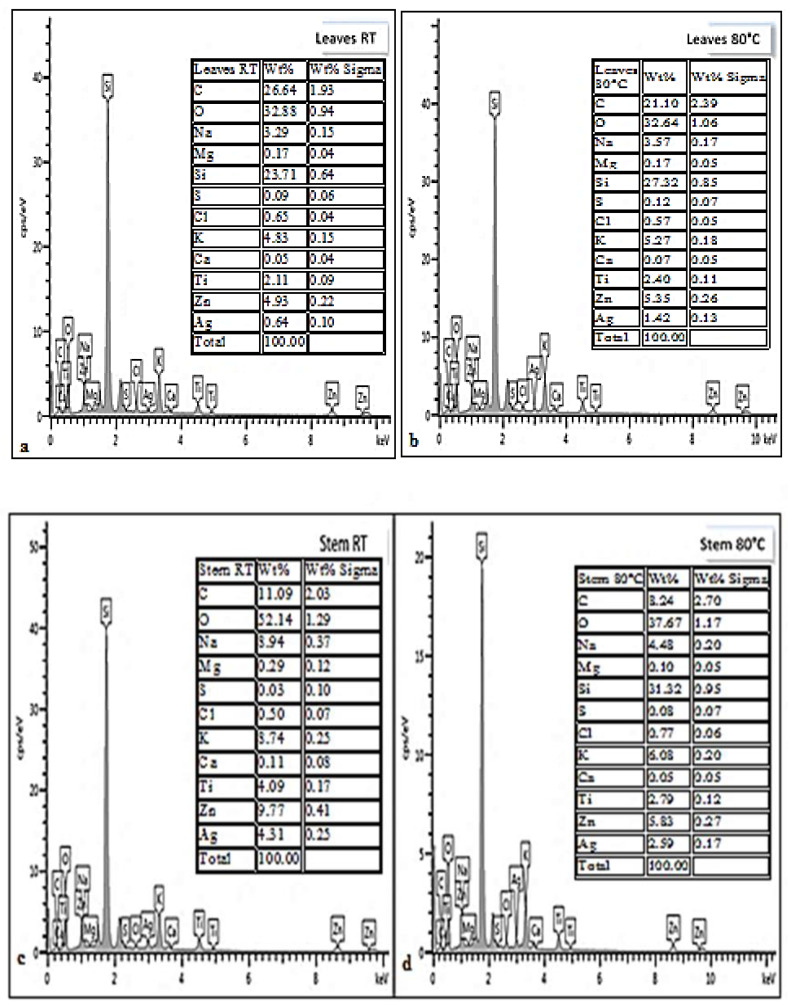
EDX spectra of *D. villosa* leaves AgNPs synthesized at room temperature (**a**), at 80 °C (**b**) and *D. villosa* stem bark AgNPs synthesized at room temperature (**c**) and at 80 °C (**d**), respectively.

EDX analysis spectra showed the existence of sodium and zinc salts within the synthesized AgNPs from *D. villosa* leaves and stem bark (Figure 5). The concentrations of both zinc and sodium salts were higher in the stem bark AgNPs synthesized at room temperature (SRT) compared to that of leaves extract AgNPs at same temperature (LRT). Other trace elements like oxygen, magnesium, potassium and carbon were further observed to exist in the *D. villosa* leaves and stem bark AgNPs. The high concentration of silicon was because of the glass slide on which the NPs were placed. Also, the Ag^+^ was quite visible at 3 keV.

### 3.6. Transmission Electron Microscopy (TEM) Analysis

**Figure 6:**
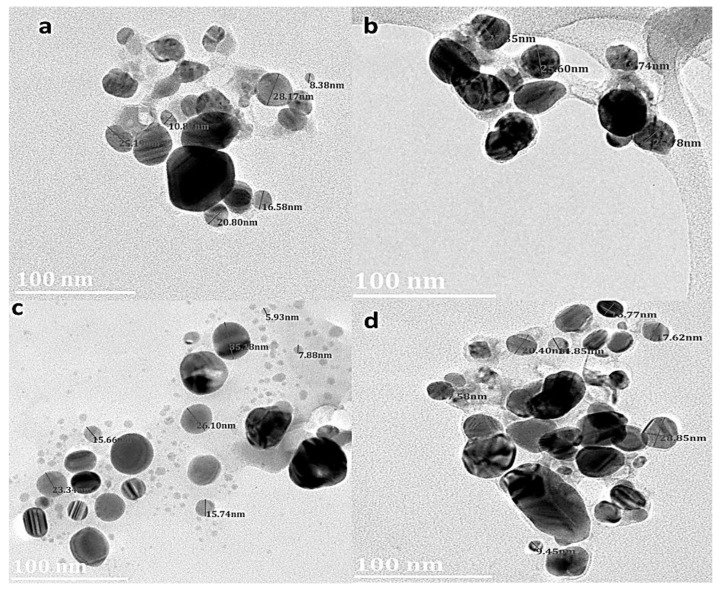
TEM images of *D. villosa* leaves AgNPs synthesized at room temperature (RT) (**a**) and at 80 °C (**c**); stem nanoparticles synthesized at RT (**b**) and at 80 °C (**d**), respectively.

The TEM images revealed different shapes and sizes of the AgNPs (Figure 6). TEM images further showed that the shape of the NPs was predominantly spherical. The diameter of the AgNPs was observed to be in the range 5 and 28 nm. Particle size distribution obtained from *D. villosa* stem bark extract AgNPs synthesized at temperature was between 19.74 and 27.85 nm. Stem bark AgNPs synthesized at 80 °C demonstrated lower sizes of ~9.45 nm.

### 3.7. DPPH Scavenging Activity

**Figure 7:**
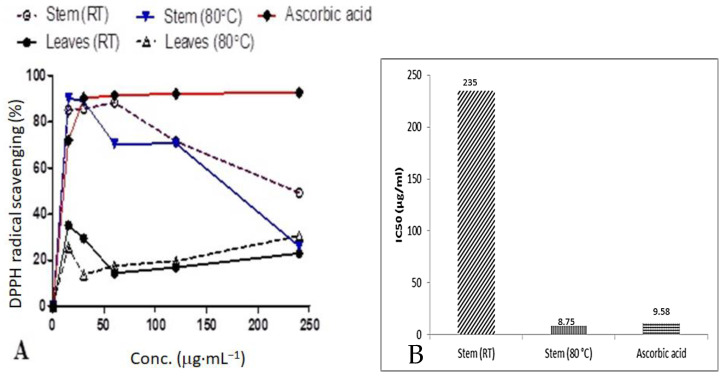
Comparison of percentage DPPH radical scavenging activities (**A**) and the IC_50_ values (**B**) of *D. villosa* leaves and stem bark AgNPs at room temperature (RT) and 80 °C.

The radical scavenging activity and IC_50_ values of *D. villosa* stem bark AgNPs are summarized in Figure 7A,B, respectively. Normally, a higher % radical scavenging activity and lower IC_50_ values indicate a higher antioxidant activity. Amongst all the synthesized NPs used in this study, stem bark AgNPs synthesized at 80 °C showed an excellent scavenging activity of 90.8%, while that of ascorbic acid at the same concentration was 72.0%. Similarly, the stem bark AgNPs of *D. villosa* synthesized at 80 °C showed an improved radical scavenging activity with an IC_50_ of 8.75 μg·mL^−1^. This value is significantly lower compared to the ascorbic acid with an IC_50_ value of 9.58 μg·mL^−1^. Meanwhile, the *D. villosa* stem bark AgNPs synthesized at RT showed weak antioxidant behavior with an IC_50_ of 235 μg·mL^−1^ compared to ascorbic acid (control). The antioxidant activity of the stem bark AgNPs synthesized at RT was higher compared to ascorbic acid, but it was not in resemblance with ascorbic acid. The same trend of higher activity was observed with leaves AgNPs, which were higher compared to ascorbic acid but with no resemblance in the antioxidant activity.

### 3.8. Ferric Reducing Antioxidant Power

**Figure 8:**
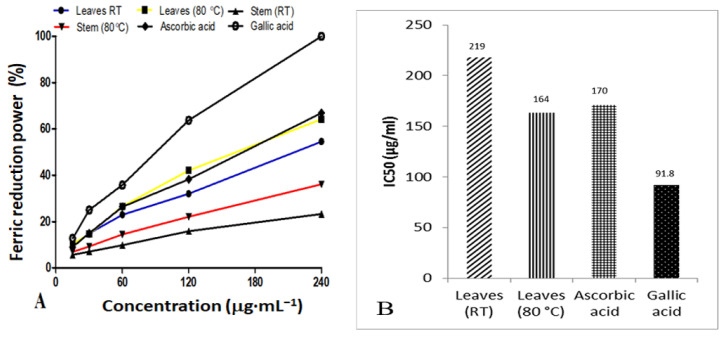
Comparison of percentage ferric reducing power (**A**) and the IC50 values (**B**) of *D. villosa* leaves AgNPs at room temperature (RT) and 80 °C.

The ferric reducing power of AgNPs synthesized from *D. villosa* leaves at RT and at 80 °C, as well as their IC_50_ values, is presented in Figure 8A,B, respectively. Generally, the IC_50_ value is inversely proportional to antioxidant activity. Based on the antioxidant results, AgNPs synthesized from *D. villosa* leaves at high temperature (80 °C) showed an effective reducing capacity with an IC_50_ of 164.0 μg·mL^−1^, which was slightly lower compared to that of ascorbic acid with an IC_50_ value of 170.0 μg·mL^−1^. However, gallic acid displayed an excellent activity with an IC_50_ value of 91.8 μg·mL^−1^. The antioxidant activity of the stem bark AgNPs was further observed to be lower compared to the control. This is evidenced by IC50 value of 8.75 μg·mL^−1^ compared to that of ascorbic acid which had a value of 9.58 μg·mL^−1^.

### 3.9. Total Phenol Content

**Figure 9:**
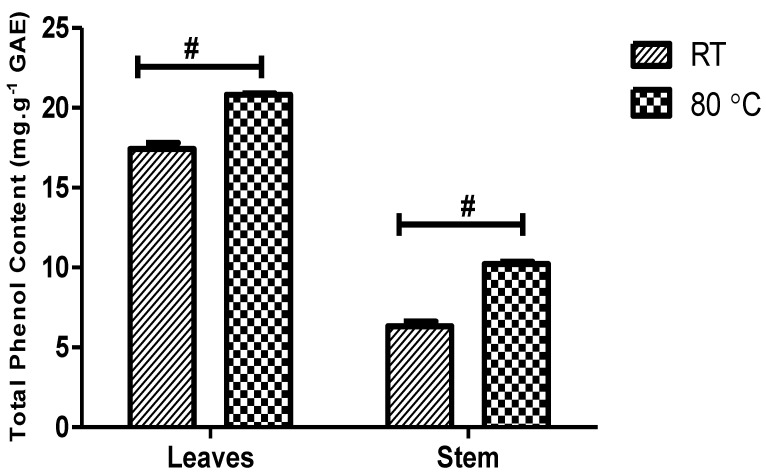
Comparison of total phenol content (mg/g) of *D. villosa* leaves and stem AgNPs at room temperature and at 80 °C. F_(1,8)_ = 219, *p* < 0.0001. ^#^ (RT vs. 80 °C), *p* < 0.001.

The total phenol content (TPC) of the *D. villosa* leaves and stem bark AgNPs was estimated and analysed (Figure 9). The TPC of the leaves and stem bark AgNPs synthesized at 80 °C was found to be 20.81 ± 0.098 and 10.22 ± 0.14 mg gallic acid equivalent per gram of dry weight, respectively. Meanwhile, the TPC of the leaves and stem bark AgNPs synthesized at RT was found to be 17.44 ± 0.36 and 6.324 ± 0.29 mg gallic acid equivalent per gram of dry weight, respectively. Two-way analysis of variance revealed that the synthesis temperature has a significant effect on TPC of AgNPs synthesized from *D. villosa* leaves and stem bark F_(1,8)_ = 219, *p* ≤ 0.0001.

### 3.10. Antibacterial Activity

In the antimicrobial susceptibility testing, stem bark AgNPs at 100 µg and 200 µg showed weak activity against both strains of *E. coli*, and *K. pneumoniae* and *P. aeruginosa* compared to the antibiotic controls (Table 2). The leaves AgNPs also demonstrated no-to-weak activity against the tested Gram-negative bacteria strains at 100 µg and 200 µg. Intermediate activity was, however, observed with the leaves AgNPs synthesized at room temperature against *E. coli* ATCC 25922 at 100 µg and 200 µg.

The stem bark AgNPs also showed weak activity against all the tested Gram-positive bacteria except against *S. epidermidis,* where intermediate activity was observed with both stem bark AgNPs at 200 µg (Table 3). The leaves AgNPs at 100 µg and 200 µg showed weak activity against *E. faecalis* ATCC 29212, *E. faecalis* ATCC 51299, *S. aureus* ATCC 29213 and methicillin-resistant *S. aureus* ATCC 43300 compared to the antibiotic controls (Table 3). However, the leaves AgNPs demonstrated intermediate activity at 100 µg and 200 µg against methicillin-resistant *S. aureus* ATCC 33591. Similarly, an intermediate activity was observed with the leaves AgNPs at 100 µg against methicillin-resistant *S. aureus* ATCC 700698 and *S. epidermis* ATCC 12228. Stronger activity was observed, however, with leaves AgNPs at 200 µg against methicillin-resistant *S. aureus* ATCC 700698 and *S. epidermis* ATCC 12228.

### 3.11. Quorum Sensing Inhibition Potential

**Figure 10:**
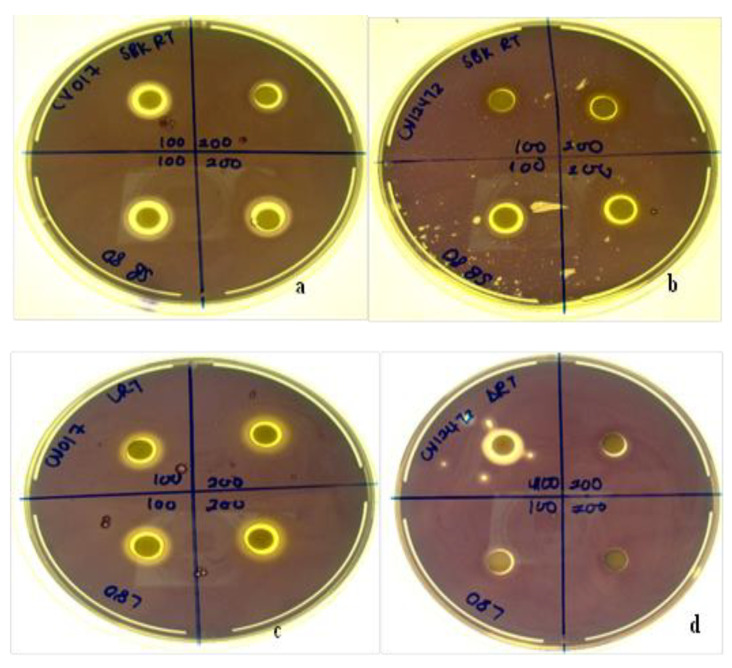
Qualitative agar overlay bioassay of *D. villosa* stem bark AgNPs synthesized at room temperature (RT) and at 80 °C using *Chromobacterium subtsugae* CV017 (**a**) and *Chromobacterium violaceum* ATCC 12472 (**b**), as well as *D. villosa* leaves AgNPs at RT and at 80 °C using *C. subtsugae* CV017 (**c**) and *C. violaceum* ATCC 12472 (**d**), displaying quorum sensing inhibition (non-pigmented zones) and antimicrobial (translucent zones) activities.

*Diospyros villosa* leaves and stem bark AgNPs synthesized at RT and 80 °C were evaluated for their QS inhibition against the *C. violaceum* ATCC 12472 and *C. subtsugae* CV017 biosensor strains. Using the agar overlay method, all NPs synthesized at both RT and 80 °C (100 µg and 200 µg) displayed QS inhibition which was indicated by the loss of purple pigment, violacein (Figure 10). Nanoparticles inhibited violacein production with QS inhibition halos ranging from 3–5 mm (Table 4). The QS inhibition appeared more predominant for *C. subtsugae* CV017 compared to *C. violaceum* ATCC 12472 (Figure 10), with both QS inhibition and halos as well as translucent zones indicating some antibacterial activity.

**Figure 11:**
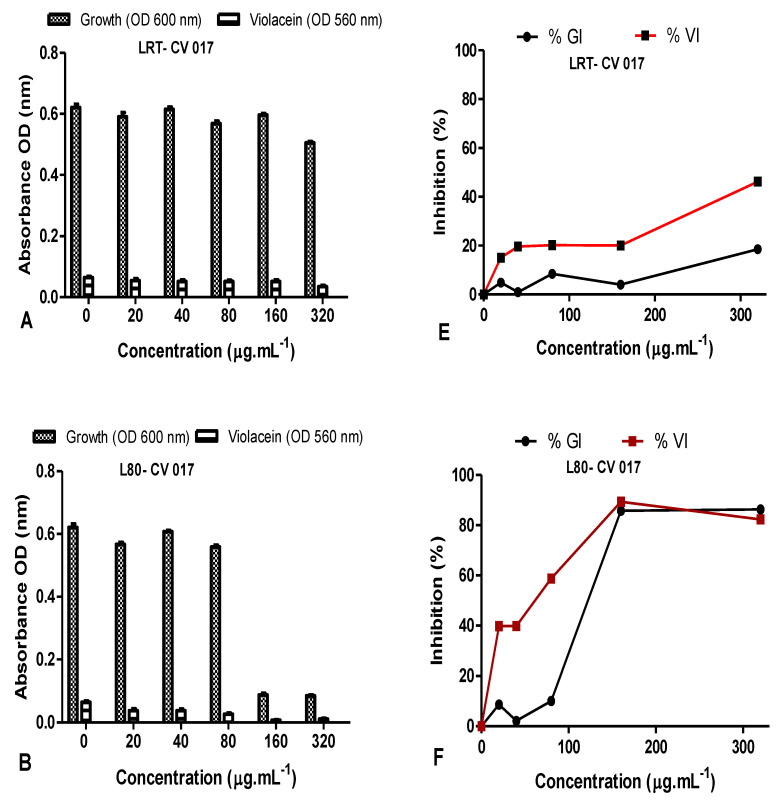

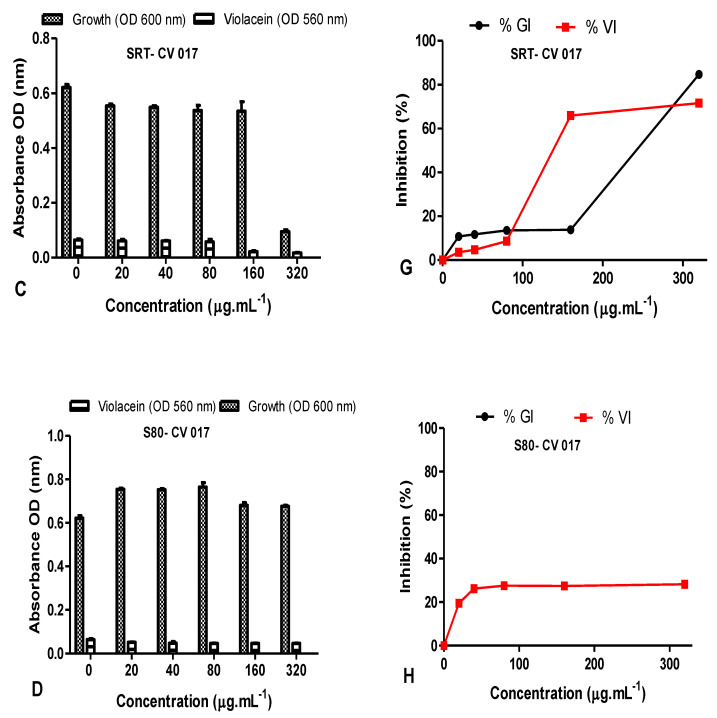
Quantitative analysis of the inhibitory effect of different concentrations of *D. villosa* leaves and stem bark AgNPs synthesized at both room temperature (RT) and at 80 °C on production of violacein by *Chromobacterium subtsugae* CV017. Cultures were grown in the presence of 0–320 µg/mL of respective leaves and stem bark AgNPs synthesized at RT and 80 °C. Frames (**A**–**D**) represent growth (OD_600_ nm) and violacein inhibition (OD_560_ nm) following nanoparticle treatment, while frames (**E**–**H**) represent %growth (%GI) and % violacein (%VI) inhibition. Data represent the average of triplicate independent experiments and SD.

The QS inhibition of the leaves and stem AgNPs synthesized at both RT and 80 °C was also quantified using *C. subtsugae* CV017 (Figure 11). The % violacein inhibition (%VI) of *C. subtsugae* CV017 by *D. villosa* leaves AgNPs synthesized at RT (LRT) were <50% at all concentrations tested (Figure 11E). However, at a concentration of 80 µg·mL^−1^, the %GI and %VI of leaves AgNPs synthesized at 80 °C (L80) were 10.08 µg/mL and 58.83 µg/mL, respectively (Figure 11B,F). Although L80 demonstrated a %VI of 89.39% and 82.27% at 160–320 µg/mL (Figure 11F), respectively, this was due to bactericidal effects, with the %GI being 85.82% and 86.28%, respectively. The %GI and %VI of *D. villosa* stem AgNPs synthesized at RT (SRT) when tested at 20–80 µg/mL were <50% (Figure 11G), however, at 160 µg/mL, the %GI and %VI values of SRT against *C. subtsugae* CV017 were 13.83% and 65.97%, respectively (Figure 11G). Bactericidal effects were observed at 320 µg/mL, with the %GI and %VI of SRT being 84.74% and 71.63% (Figure 11G). *Diospyros villosa* stem bark AgNPs synthesized at 80 °C (S80), in contrast, showed negligible %GI with %VI < 30% at all the concentrations tested (Figure 11H).

**Figure 12:**
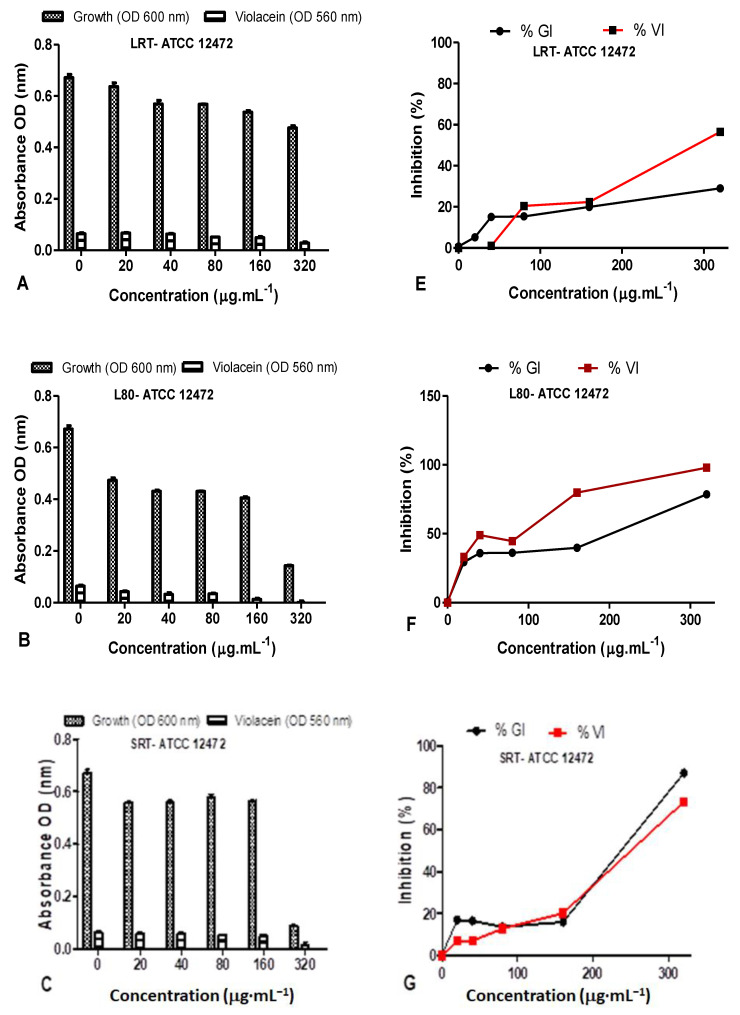

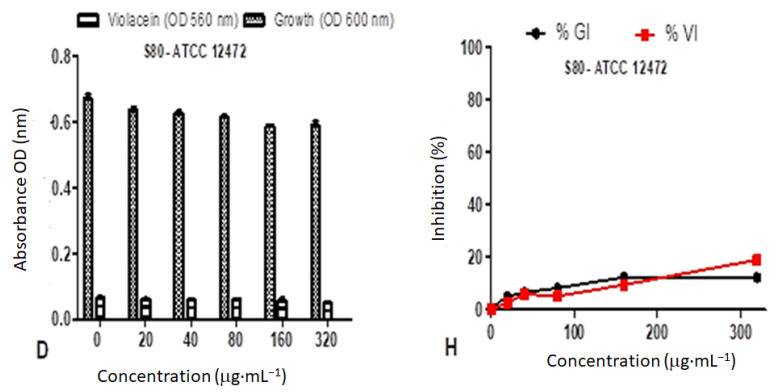
Quantitative analysis of the inhibitory effect of different concentrations of *D. villosa* leavesAgNPs synthesized at both room temperature (RT) and at 80 °C (80) on production of violacein by *Chromobacterium violaceum* ATCC 12472. Cultures were grown in the presence of 0–320 µg/mL of respective leaves nanoparticles at RT and 80 °C. Frames (**A**–**D**) represent growth (OD_600_ nm) and violacein inhibition (OD_560_ nm) following nanoparticle treatment, while frames (**E**–**H**) represent %growth (%GI) and % violacein (%VI) inhibition. Data represent the average of triplicate independent experiments and SD.

The QS inhibition of the leaves AgNPs synthesized at both RT and 80 °C were quantified using *C. violaceum* ATCC 12472 as the indicator biosensor (Figure 12). The %GI and %VI of *D. villosa* LRT against ATCC 12472 was <50% at 20–160 µg/mL (Figure 12C), however, at 320 µg/mL, the %GI and %VI of LRT against *C. violaceum* ATCC 12472 was 29.07% and 56.53% (Figure 12C). The %GI and %VI of L80 nanoparticles against *C. violaceum* ATCC 12472 at 20–80 µg·mL^−1^ was <50% (Figure 12D), however, bactericidal activity was observed from 160–320 µg/mL (Figure 12D).

The %GI and %VI of *D. villosa* leaves AgNPs synthesized at 80 °C (S80), against *C. violaceum* ATCC 12472 at the concentration of 320 µg/mL was observed to be 78.53% and 97.97%, respectively (Figure 12D). The LRT at a concentration of 320 µg/mL may rather be considered a good antimicrobial agent compared to QS inhibitor. The *D. villosa* leaves AgNPs synthesized at 80 °C (S80) demonstrated %VI of <50% at tested concentrations below 100 µg·mL^−1^ against ATCC 12472 (Figure 12H).

## 4. Discussion

The NPs used in this study were synthesized from *D. villosa* plant leaves and stem bark extracts with the reaction temperature being varied, i.e., room temperature vs. 80 °C. Distilled water was used as the reactional medium while the plant metabolites were used as the reducing and capping agents. The *D. villosa* leaves and stem bark AgNPs were characterized and investigated for their antioxidant, antibacterial and QS inhibitory activities. The choice of temperature clearly affected the percentage yield of NP especially with the leaves extracts (Figure 1). This result is in support of Dhanani et al. [43], where the composition of plant extracts is dependent on the extraction temperature. Higher temperature enhances the extraction due to lowered solvent viscosity and higher solute solubility. Furthermore, a moderately high extraction temperature could be adopted to enhance the maximum extraction of the targeted compounds which are further responsible for reduction and capping of the NPs.

The appearance of the brown color was an indication of the silver ionic reduction and formation of AgNPs. This is also in accordance with Zayed et al. [44], where it was reported that bioreduction of a silver ion was catalyzed by the reducing metabolites present in the extract. This photosynthetic observation was further monitored by UV-visible spectroscopy. The absorption spectra showed that the surface plasmon resonance (SPR) band for the *D. villosa* leaves at both temperatures (RT and 80 °C) matched that of conventional AgNPs. This observation confirms previous findings of Baruah et al. [45], where the SPR band for AgNPs was around 420 nm and that the SPR peaks of NPs arise due to the motion of the metal free electrons in resonance with the irradiated light. The position of the free electron depends on the size and shape of the AgNPs [45]. The presence of single SPR is also a clear indication that the biosynthesized AgNPs from the leaves extracts of *D. villosa* are significantly likely to be spherical in shape which is further confirmed by both SEM and TEM analyses.

SEM showed the spherical shape of the NPs, but better morphological characterization of NPs was achieved using TEM. Transmission electron microscopy provided evidence that the NPs were predominantly spherical in shape; less agglomerated and distributed particularly in the temperature enhanced biosynthesized AgNPs (Figure 6C,D). The difference in the degree of agglomeration in the temperature-enhanced biosynthesis as compared to that of room temperature was suggested to occur due to the variety of bio-reducing compounds. Vinod et al. [46] suggested that the NPs have a propensity to aggregate together forming micro sized particles that are more stable. The nature of the biomolecules present in the *D. villosa* leaves and stem bark was proven by FT-IR analysis (Figure 3). From our results, it can be deduced that the observable bands at 3369 cm^−1^ could be ascribed to hydroxyl stretching vibrations. The same specified range was assigned to O-H stretching vibration, with further explanation that the O-H are bonded to sites through strong bond interaction [47]. Hence, the characteristics of O-H stretching, as shown with the absorption peak at 3369.91 cm^−1^, further suggested the presence of aromatic acids [48]. Also, the spectral analysis depicted that the O-H group in the AgNP biosynthesized extract of *D. villosa* may likely be involved in the reduction of silver ions. The biological molecules of the extract, especially the hydrogen contents, could act as the reducing agent for the AgNPs. Similarly, the observable bands at 1620 cm^−1^ were assigned to C=O aromatic vibrations. These results confirm FTIR data reported by Tarekegne et al. [49], where the peak at the same range with our observable value was attributed to symmetric C=O stretch. There is no major difference in the embedded functional groups of the biomolecules when considering the biosynthesis of AgNPs using temperature variation.

Antioxidants are known to defend living cells against harmful effects incurred through oxidative stress [50]. The biosynthesized *D. villosa* leaves and stem bark AgNPs showed free radical scavenging properties, but to varying degrees. The AgNPs synthesized at 80 °C demonstrated better DPPH antioxidant activity compared to AgNPs synthesized at RT. Both the leaves and stem AgNPs synthesized at 80 °C had the best antioxidant activity among all the four AgNPs tested (Figure 7 and Figure 8). With IC_50_ of 8.75 and 164.0 μg·mL^−1^ using DPPH and FRAP, respectively. The results were comparable to that of ascorbic acid positive control (9.58 and 170 μg·mL^−1^) respectively. This is consistent with the findings of Bizimenyera [51]. An extensive review of medicinal plants with potent antioxidant activities by Atawodi [52] indicated that the mechanism of action was specifically free radical scavenging. In addition, the synergistic effect of embedded biochemicals enhance their antioxidant activity [53] also found that AgNPs synthesized from *D. montana* leaves extracts demonstrated higher DPPH scavenging activities compared to the crude extracts. This result also supported the DPPH radical scavenging assays that had been reported for AgNPs synthesized under enhanced temperature. In addition, phenols are recognized as an essential bioactive compound because of their capacity as an antioxidant [54]. Therefore, the presence of a hydroxyl ion, as shown in the FT-IR spectral analysis, could be another reason for the effective antioxidant capacity of the nanoparticles. Thus, *D. villosa* extracts can be useful to produce AgNP antioxidants with safe biomedical and healthcare applications.

Natural product antimicrobial activity is thought to be noteworthy if the inhibitory potential is demonstrated against either Gram-negative and/or Gram-positive bacteria [55]. The leaves NPs (LRT and L80) indicated potency for antibacterial activity. Only two pathogens, *S. aureus* ATCC 700698 and *S. epidermidis* ATCC 12228, were susceptible to the leaves AgNPs (zone of inhibition = 16 mm) at 200 µg. Methicillin-resistant *S. aureus* ATCC 700698 was also susceptible to the L80 AgNPs with an inhibition zone of 18 mm. Gram-positive bacteria were more vulnerable to the AgNPs synthesized from *D. villosa* leaves and stem bark. The leaves AgNPs displayed strong antibacterial activities against *S. aureus* and *S. epidermidis,* suggesting that leaves AgNPs may have applications in topical ointments as a curative measure to *S. epidermidis* and *S. aureus* skin infections. The antimicrobial activity of some plants within the same genus as *D. villosa* has been reported against various pathogens [53,56]. It has been reported that NPs synthesized from *D. malabrica* resulted in diminished bacterial growth against *E. coli* and *S. aureus,* respectively [57]. This agrees with the obtained results in this study, as the LRT revealed potent bactericidal activity against *E. coli* ATCC 25922, *S. aureus* ATCC 33591, ATCC 700698 and *S. epidermidis* ATCC 12228. The L80 NPs also demonstrated bactericidal activity against *S. aureus* ATCC 700698 and *S. epidermidis* ATCC 12228. The slight variation observed in the NP activity, as reflected in the result of leaves nanoparticles, is corroborated by the reports of Gontijo et al. [58], with some selected NPs having non-significant activities while others yielded better activities. The varying degrees in bioactivity may be due to the interplay of secondary metabolites from extracts [59]. When QS inhibition against the short-chain acyl homoserine lactone (AHL) producer *C. subtsugae* CV017 was assessed, the L80 and SRT AgNPs demonstrated violacein inhibition at 80 and 160 µg/mL of 58.83 and 65.97%, respectively. This inhibition was accompanied by bactericidal activity at the higher concentrations tested. By contrast, when QS inhibition against long-chain AHL *C. violaceum* ATCC 12472 was assessed, only the LRT AgNPs exhibited violacein inhibition of 56.53% against *C. violaceum* ATCC 12472 at 320 µg/mL (Table 3).

Traditional medicinal plants have been proven to interact with bacterial quorum sensing and attenuate pathogenicity of bacteria [60]. There are a limited number of studies on the anti-quorum sensing activity of plant synthesized AgNPs [17,61,62] using *Chromobacterium* as an indicator of QS inhibition. Although the L80 and SRT AgNPs demonstrated short chain AHL inhibitory potential, the inhibition ranged from 58.83–65.97% at 80 and 160 µg/mL, respectively, while only the LRT AgNPs inhibited the long chain AHL-producing *C. violaceum* ATCC 12472 at 320 µg/mL. This is much lower than the inhibition obtained by Shah et al. [61], who obtained 72.25–97.48% inhibition using 5–20 µg/mL of *Piper betle* AgNPs, while Qais et al. [62] obtained 34.2–74.7% inhibition using 1–4 µg/mL of *Carum copticum* AgNPs. The *D*. *villosa* AgNPs demonstrated QS inhibition at lower concentrations but were bactericidal at the higher concentrations of 160–320 µg/mL, with growth inhibition ≥ 40% indicated bactericidal activity. This was also observed by Ali et al. [17], who observed both QS inhibition and bactericidal activity in the qualitative agar-based assay. The *D. villosa* AgNPs were not as potent as the respective plant extracts (data not shown) in terms of QS inhibition. It is promising to note that the leaves AgNPs have demonstrated potential as antimicrobial agents against Gram-positive bacteria, as well as some promising QS inhibition of Gram-negative bacteria.

## 5. Conclusions

A suitable and more reliable antioxidant potential was obtained with temperature enhanced AgNPs. Furthermore, temperature enhancement of AgNP biosynthesis facilitates a more distinct size of *D. villosa* stem bark NPs and with better potency as a reliable antioxidant. Also, this experimental research study was done to determine the QS inhibitory potential of *D. villosa*. The promising QS inhibitory and antimicrobial bioactivities of the *D. villosa* stem NPs suggest that optimization is required to obtain greater antimicrobial and/or QS inhibitory activity.

## Figures and Tables

**Table 1:** The synthesized AgNPs using *D. villosa* leaves and stem bark at different temperature ranges (25 °C and 80 °C).

Temperature (°C)	Part of the Plant	
	Leaf (L)	Stem Bark (SB)
25 °C (RT)	LRT	SBRT
80 °C	L80	SB80

**Table 2:** Zone of inhibition (mm) of *D. villosa* leaves and stem AgNPs against Gram-negative bacteria strains.

AgNPs	*E. coli* ATCC 25922		*E. coli* ATCC 35218		*K. pneumoniae* ATCC 700603		*P. aeruginosa* ATCC 27853	
	100 μg	200 μg	100 μg	200 μg	100 μg	200 μg	100 μg	200 μg
L RT	11	15	7	8	0	0	0	0
L 80	8	9	7	9	0	0	0	0
SB RT	0	8	7	9	8	8	8	10
SB 80	0	9	7	8	8	8	8	10
Control								
CIP5	30 (S)		37 (S)		26 (S)		32 (S)	
GN10	19 (S)		20 (S)		17 (S)		19 (S)	

Weak ≤ 10 mm; Intermediate (11–15) mm; Strong ≥ 16 mm. CIP5: Ciprofloxacin; GN10: Gentamicin; LRT: Leaves AgNPs synthesized at room temperature. L80: Leaves AgNPs synthesized at 80 °C; SB RT: Stem bark AgNPs synthesized at room temperature. SB 80: Stem bark AgNPs synthesized at 80 °C. Response to tested compounds is indicated by S (sensitive) and R (resistant).

**Table 3:** Zone of inhibition (mm) of *D. villosa* leaves and stem AgNPs against Gram-positive bacterial strains.

AgNPs	*E. faecalis* ATCC 29212		*E. faecalis* ATCC 51299		*S. aureus* ATCC 29213		*S. aureus* ATCC 33591		*S. aureus* ATCC 43300		*S. aureus* ATCC 700698		*S. epidermidis* ATCC 12228	
Quantity (µg)	100	200	100	200	100	200	100	200	100	200	100	200	100	200
L RT	9	10	8	9	9	9	12	14	9	9	11	16	12	16
L 80	9	10	8	9	8	7	11	14	8	8	12	18	12	14
SB RT	7	8	0	8	0	8	0	8	7	8	0	8	9	12
SB 80	8	9	0	9	0	10	8	10	0	7	0	10	8	11
CIP5	33 (S)		38 (S)		23 (S)		22 (S)		23 (S)		6 (R)		28 (S)	
GN10	18 (R)		0 (R)		19 (R)		16 (S)		9 (R)		11 (R)		20 (S)	

Weak ≤ 10 mm; Intermediate (11–15) mm; Strong ≥ 16 mm. CIP5: Ciprofloxacin; GN10: Gentamicin. LRT: Leaves AgNPs synthesized at room temperature. L80: Leaves AgNPs synthesized at 80 °C; SB RT: Stem bark AgNPs synthesized at room temperature. SB 80: Stem bark AgNPs synthesized at 80 °C. Response to control antibiotics is indicated by S (sensitive) and R (resistant).

**Table 4:** Zone of inhibition (mm) of *D. villosa* leaves and stem bark AgNPs against *C. subtsugae* and *C. violaceum* biosensors.

AgNPs	*C. violaceum* ATCC 12472						*C. subtsugae* CV017					
	100 μg			200 μg			100 μg			200 μg		
	TZD	CZD	QSI	TZD	CZD	QSI	TZD	CZD	QSI	TZD	CZD	QSI
L RT	13	8	5	14	9	5	13	8	5	14	9	5
L 80	12	8	4	13	8	5	13	8	5	14	9	5
SB RT	11	7	4	14	9	5	13	10	3	14	9	5
SB 80	12	8	4	15	10	5	15	11	4	14	9	5
Vanillin	10	0	10	16	0	16	10	0	10	15	0	15

TZD = Total zone diameter in mm. CZD = Clear, bactericidal zone diameter in mm. QSI = Quorum sensing inhibition, opaque zone in mm. LRT: Leaves AgNPs synthesized at room temperature. L80: Leaves AgNPs synthesized at 80 °C; SB RT: Stem bark AgNPs synthesized at room temperature. SB 80: Stem bark AgNPs synthesized at 80 °C.

## Data Availability

Not applicable.
